# News analysis

**DOI:** 10.1136/tc.2009.033167

**Published:** 2009-09-17

## BRAZIL: SÃO PAULO TAKES THE LEAD

At exactly 12:01 AM on 7 August, Brazil’s most populous state, São Paulo, became smoke-free. The law went into effect without any problems and compliance was high in the first few hours, with only isolated reports of non-compliance—just 11 fines out of 887 establishments inspected. The law requires all workplaces, including bars, restaurants and night clubs, to be 100 per cent smoke-free, going beyond existing federal legislation dating from 1996. In addition, it applies to common areas of apartment buildings. The only exception is for religious cults in which tobacco might be an essential part of the rituals.

The system of fines is progressive and leaves no doubt that the law means business. Initial fines are between R$792.50 to R$1,585.00 (approximately US$435 to US$870), depending on the size of the establishment, for a first offence, which includes the presence of ashtrays. The fine is doubled for a second offence. After a third offence, the establishment has to close for 48 hours; and after a fourth, it must close down for 30 days.

All articles written by David Simpson unless otherwise attributed. Ideas and items for News Analysis should be sent to: d.simpson@iath.org

The law was drafted by the state’s Governor, José Serra, the former federal health minister responsible for Brazil's pictorial health warnings and advertising restrictions. The parliamentary debate in the State Assembly was like a parody of tobacco industry tactics, with hospitality associations with known links to tobacco companies claiming that the proposed legislation was unconstitutional, that economic disaster would ensue, and that the rights of smokers would be violated, among other well-known industry arguments. Advocacy groups, particularly Alianca de Controle do Tabagismo (ACT), together with public health professionals, mounted a well organised educational and media campaign to defeat the industry’s arguments. The assembly approved the legislation in April and it was duly signed by the Governor in May, with three months of educational campaigns for businesses and the public to prepare.

Shortly after approval, a flood of lawsuits were initiated by various hospitality associations in several cities. The state government has responded to all of them, refusing to agree to exceptions or designated smoking rooms. So far the courts, either initially or on appeal, have found in favour of the government. Most importantly, none of the pending cases stopped the law from going into effect.

Public opinion polls continue to demonstrate overwhelming support for the legislation and courtesy visits by inspectors confirmed that more than 80 per cent of businesses were in compliance weeks before the 7 August implementation date. In preparation, the state government created a media campaign, including countdown clocks and giant hourglasses, located in several main areas of the capital, São Paulo, and other major cities. It also created a website and communicated with the public via social networking internet sites and Twitter.

With a state population of more than 40 million, São Paulo's smoke-free legislation is among the most far-reaching in the world in terms of the number of people protected from exposure to second-hand smoke. There are high hopes that other states and large cities in Brazil will follow this example.

STELLA AGUINAGA BIALOUS

San Francisco, USA

stella@bialous.com

STELLA MARTINS

São Paulo, Brazil

**Figure:**
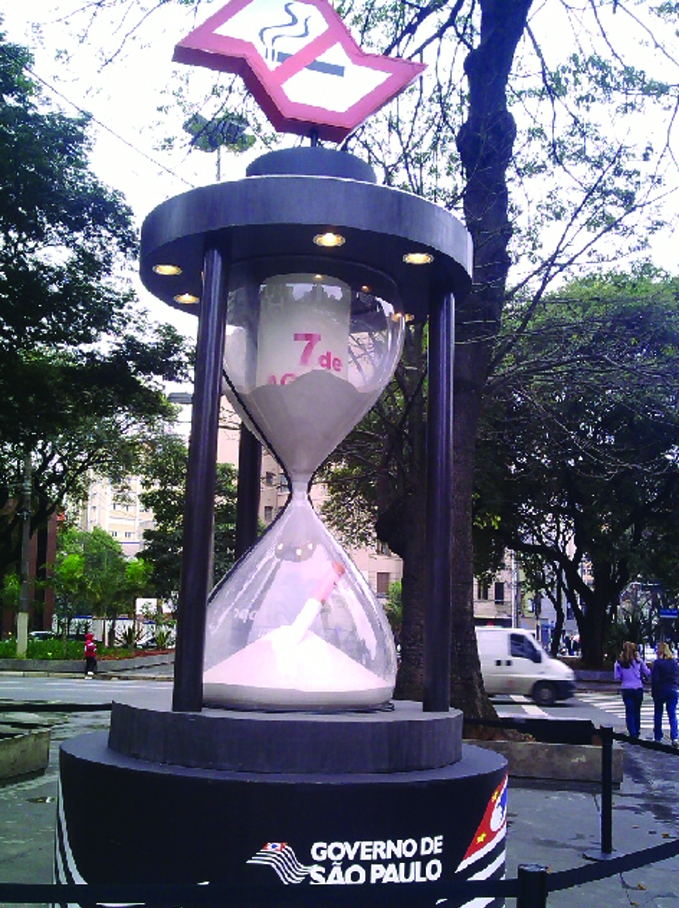


**Figure:**
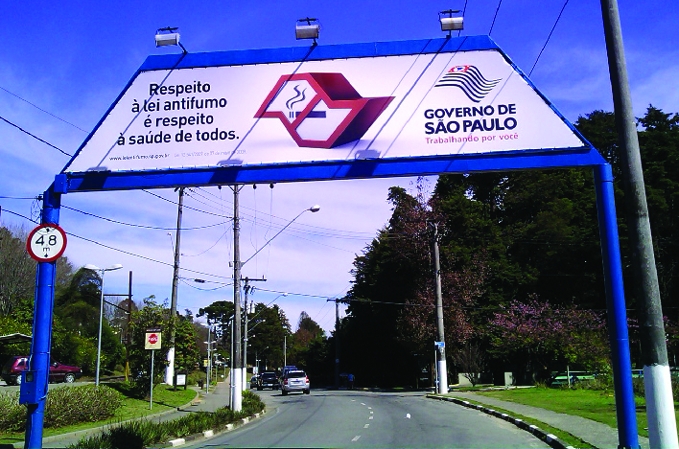
Brazil: signs in parks, over roads and in other public areas notified São Paulo state’s 40 million citizens of the deadline for the ban on smoking in public places. *[Photos: Stella Martins]*

## WORLD: NEW DATA, HARD SLOG AT INB3

The feeling among non-governmental organisations at the close of the third intergovernmental negotiating body (INB3) sessions held in Geneva, Switzerland in early July was that real progress had been made, but there was still a very great deal left to be accomplished. INB negotiations develop detailed guidelines for the implementation of the Framework Convention on Tobacco Control (FCTC) and INB3 sought common ground for developing a protocol on illicit trade in tobacco products.

Several important new pieces of research were released to coincide with the negotiations, including the most authoritative report yet produced on the extent of the global illicit trade in cigarettes. Published by the International Union Against Tuberculosis and Lung Disease and compiled by an international team of experts, the report, How Eliminating the Global Illicit Cigarette Trade would Increase Tax Revenue and Save Lives, provided delegates with updated country-level estimates of the illicit cigarette market around the world, using the most recent data available, mostly from 2007. It showed that higher income countries, where cigarettes are more expensive, have lower levels of cigarette smuggling—9.8 per cent—than lower income countries—16.8 per cent—contrary to the tobacco industry’s mantra that the overall level of smuggling is dependent on cigarette price.

The new report also showed that the burden of cigarette smuggling falls disproportionately on low- and middle income countries, where the majority of the world’s tobacco users live; and estimated the number of lives saved and revenue gained globally in the future if smuggling were eliminated. It showed that 11.6 per cent of the global cigarette market is illicit, equivalent to 657 billion cigarettes a year and US$40.5 billion in lost revenue—substantially higher than the 2000 World Bank estimates, based on 1995 data, of 6 to 8.5 per cent of global cigarette consumption. If illicit trade were eliminated, governments would gain at least US$33 billion per year, and from 2030 onwards more than 160,000 lives a year would be saved, the report concluded.

The negotiations were complex and difficult, often spilling over into the lobbies, sometimes late into the night, as attempts were made to get informal agreements on individual topics before the next session. By the end, the interim text was rated highly unsatisfactory by most, but at least some progress had been made as a basis for the next round of work. So while the Parties—governments that have both signed and ratified the FCTC—came nowhere near agreeing upon a final draft protocol, they did at least leave Geneva with a clearer idea of why other Parties were taking their respective positions.

Now it is up to smaller groups to thrash out the details before the next full negotiations, INB4, which take place in Geneva during the first quarter of 2010. Among the most critical issue to be resolved is tracking and tracing tobacco products, an area of considerable disagreement, especially as one proposal, the existing European Union policy, seems all too close to one favoured by Philip Morris International. Clearly the task ahead, like the range of differences between Parties, is huge. Nevertheless, most observers believe that INB4 could still deliver a Protocol that would be a significant step in the right direction.

## GERMANY: TOBACCO ATLAS, AT LAST

In July, the first German tobacco atlas was published by Deutsches Krebsforschungzentrum (DKFZ), the German cancer research centre. The very fact of publication, the way it was done and not least, the comprehensive contents of the new publication are testimony to the extraordinary, if long overdue, progress being made in the country once dubbed the tobacco industry rent-a-nation of the European Union.

For one of the most scientifically and technically advanced countries in the world, it had long been surprising how little action had been taken in Germany to reduce the huge burden to health caused by tobacco and how vigorous and apparently free news media could consistently ignore the problem. Germany entered the 21st century with a great deal of catching up to do (see Germany: how did it get like this? *Tob. Control*, Dec 2002;**11**:291–3).

At last, however, the tide has well and truly turned. What seemed in the past like an unofficial media embargo on reporting anything even mildly critical of tobacco or the tobacco industry is now just a bad memory, as evidenced by exceptionally wide and serious coverage of the atlas.

Two thirds of the 130-page atlas is devoted to a detailed description of the tobacco problem. In addition to the dangers of active and passive smoking, this includes regional data on smoking prevalence, social inequalities, regional death rates from the main tobacco induced diseases, the costs of smoking, additives, the problems with “harm reduction” approaches and facts about the tobacco industry, this last accounting for 13 pages alone. The final third of the book is devoted to tobacco control policy, tightly referenced to the World Health Organization's Framework Convention on Tobacco Control (FCTC), to which Germany is a party, linking relevant FCTC articles directly to the German situation.

In addition to the impact of its factual contents, the atlas is a model of good design and clear presentation, which may partly account for some of the enthusiastic comments received from individual journalists. One, for example, recounted how she had read right through the entire book, page by page, because she had been so fascinated by the facts it presented, adding that she had found it "like reading a crime story" — exactly the sort of epiphany that DKFZ has been working for years to provoke among its country's media and politicians.

Even the manner of publication broke new ground. In the past, serious publications about tobacco, even from a reputable, state-sponsored cancer research organisation, appeared to receive little attention from the media, politicians or government. This time, however, Sabine Bätzing, drugs commissioner within the ministry of health, offered the resources of her office to launch the atlas. It was duly released with Ms Bätzing present, at a press conference in the federal press conference room to which all German media have access, a most unusual privilege. This resulted in extensive coverage in media ranging from national radio and television channels to *Spiegel*, the highly influential (and once tobacco-blind) news magazine, and online political magazines. More than 100 press reports appeared in total, covering all regions of Germany.

In addition to focussing on the north-south tobacco divide in Germany—with the smoky, less affluent north contrasting with the lower smoking, richer south—the responsibility of the tobacco companies was fingered by many commentators. The director of the German Cancer Research Centre, Dr Otmar Wiestler, told the press conference that he could not understand how so many deaths had been met by so little political will to implement stronger tobacco control legislation, which he concluded was due to the continuous efforts of tobacco lobbyists. *Spiegel* printed an interview with Dr Wiestler in which he specifically criticized the tobacco industry’s influence in German politics.

Perhaps most telling was the coverage in *Die Tabak Zeitung (DTZ)*, the weekly publication of the German tobacco industry. Unable to write off a clearly important, evidence-based publication about its product, one with government backing that was attracting so much interest in the general media, *DTZ* assumed a somewhat contradictory position. On the one hand, it said, most parts of the atlas were absolutely serious, but the selection of material was tendentious and the interpretation often polemic. In short, *DTZ* concluded, reaching for the somewhat archaic stereotype of China's late Chairman Mao Zedong and his “little red book”, the atlas was a “Mao bible” of tobacco opponents.

With such a reaction from the industry, and enthusiastic backing form the health ministry, DKFZ has clearly presented a superb, ground-breaking new resource. The next stage will be all important: the government must now follow the steps so clearly set out in the atlas to fully implement the FCTC and put Germany on track to tackle its massive tobacco problem. [Tabakatlas Deutschland 2009 can be downloaded at www.tabakkontrolle.de and further information is available at www.drogenbeauftragte.de]

**Figure:**
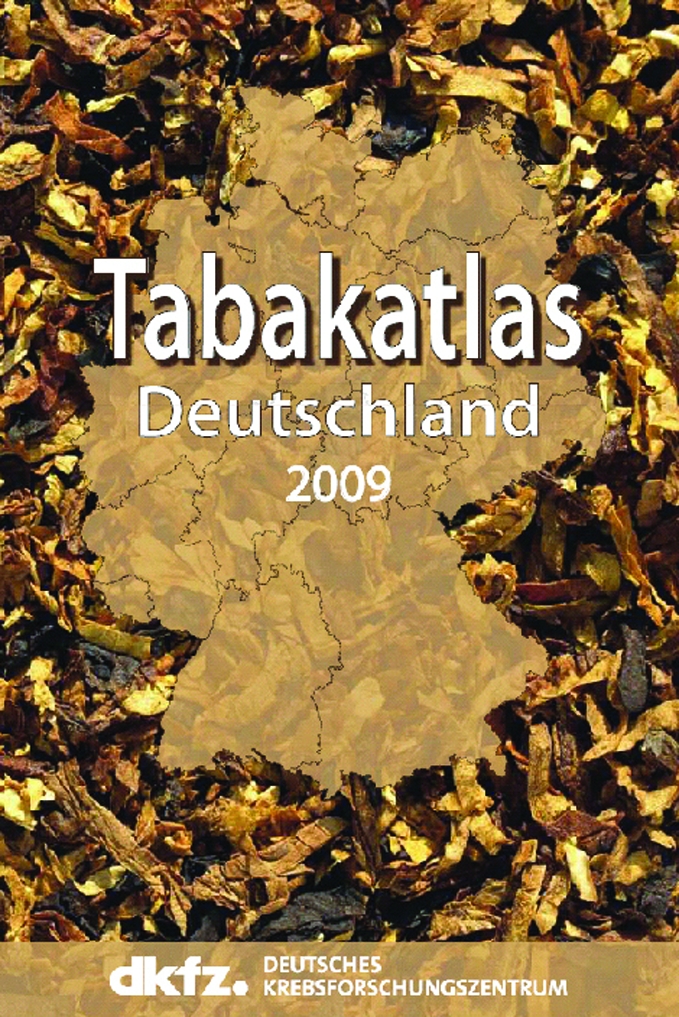


## INDIA: BID TO RAISE GUTKHA WARNING

So many different products containing tobacco are consumed in India—more than 20, in fact—that just the text of a website attempting to briefly describe them all would take up more than three full pages of this journal. Apart from smoking it, Indians consume it, knowingly or not, by sucking or chewing it, taking it as snuff in either the nose or the mouth, and even in toothpaste. In stark contrast to most other countries, more premature deaths are caused by oral tobacco products than by tobacco smoking in India.

No wonder, then, that those who daily try to mitigate, heal or repair the damage done by oral tobacco should be trying to raise awareness of it. In particular, one product is so socially acceptable that in many areas its use is as prevalent among children as it is in adults. Gutkha, a powdery or granular preparation of betel nut, tobacco, lime and savoury flavourings, is among the most commonly used of all India's oral tobacco products. Originally a moist preparation, since the 1990s Indian tobacco companies have been selling a dried version in smart pouches and sachets.

With euphemisms and pseudo-science reminiscent of those used by cigarette makers, one manufacturer boasts, “We manufacture a wide range of regular and premium gutkhas widely acclaimed in the masses and the connoisseur class for their rich aroma and relaxing effect. Our gutkhas are manufactured from natural elements easily accepted by human systems and does not affect the functioning of their systems.” Many manufacturers market gutkha to children by pitching it as confectionery or mouth freshener and some packs highlight the chocolate or mint flavours of their contents. Most do not draw attention to the inclusion of tobacco, or if they do, fail to specify the concentration.

Little wonder, then, that many school children think of gutkha simply as a harmless mouth freshener. To make matters worse, many consume it in large amounts and keep it in the mouth for a long time. So little wonder, either, that Dr Pankaj Chaturvedi, the oral surgeon from Mumbai whose creative innovations in tobacco control have featured in past editions of this journal (eg, *Tobacco Control* 2009;**18**:163–4), should seek to elevate his profession’s awareness of this particular health hazard. In fact, gutkha deserves a double warning, as areca, the nut of the betel palm used in gutkha and other chewing products in India, is itself carcinogenic.

Dr Chaturvedi reports that as an oral surgeon, he sees several chewers of gutkha and similar products every day in his clinic and over the years has found a striking pattern in the signs and symptoms of these chewers. In response, as one of a range of activities to highlight gutkha’s harmfulness, he has launched a campaign to establish professional acceptance of “gutkha syndrome” and the consequences he has identified among habitual users. His descriptions of gutkha syndrome have been published in several medical and dental journals. In the *British Dental Journal* in April, for example, he listed the various components of the syndrome, caused by fibrosis in the sub-mucosal layers and in the muscles of mastication, affecting the appearance of the face, mouth, gums and teeth, the quality of speech, swallowing ability and hearing impairment. He also warned that the consumption of gutkha or similar products had not only been reported from Asia but also from the western world, especially where significant Indian populations have settled.

Dr Chaturvedi has also created a website, Arecapedia, as “an attempt by health professionals to educate people about the areca nut and its harmful health consequences.” It is accessible via the Google search engine (http://sites.google.com/site/quitnut/Home/areca-syndrome).

In recent years India has made great strides to enact and implement health policies to deal with tobacco smoking, far exceeding what seemed possible to visiting experts from overseas as little as just two decades ago. However, the oral tobacco problems which such visitors heard described at cancer meetings in those dark days still look doggedly persistent and unmatched by the level of awareness and action that is nowadays expected for smoking. Promotion of some of the culprit products still continues in ways that would now be unacceptable or illegal, as well as banned for cigarettes. It remains to be seen whether the efforts of Dr Chaturvedi and others can help to redress the balance.

**Figure:**
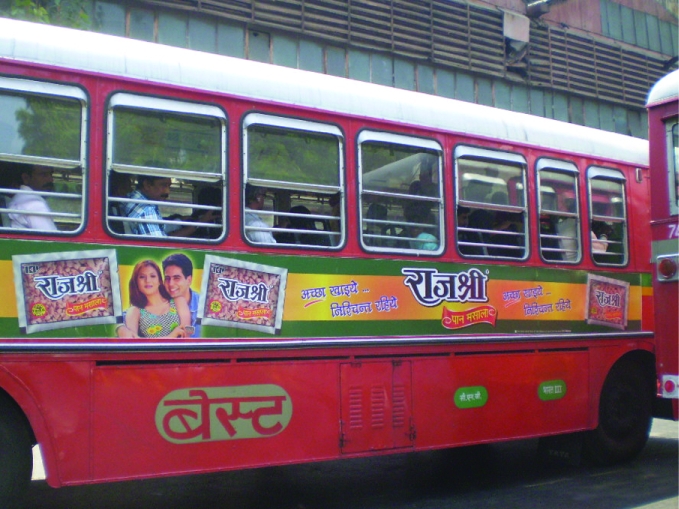
India: a gutkha advertisement on a bus.

## GREECE: SMOKING BAN, OR SMOKE & MIRRORS?

Greece is one of the heaviest smoking nations in the world and given current trends, unless effective preventive action is taken, smoking rates will continue to increase. Almost three years after the ratification of the World Health Organization’s Framework Convention on Tobacco Control (FCTC), Greece is implementing a new tobacco control policy. From July this year, a new law has come into force to prevent exposure to second-hand smoke (SHS) in public settings, such as restaurants, cafeterias, bars, and clubs. While it is hoped that the new policy represents progress towards preventing SHS exposure in a heavy-smoking culture, there are serious concerns that the policy’s effectiveness may be undermined.

Firstly, the law allows the owners of leisure venues with a usable space smaller than 70 square metres to choose between a total smoking ban and no ban at all. There is no clear rationale for allowing owners of smaller establishments to make this choice; indeed, it might be expected that smaller establishments, where even the (ineffective) option for separate smoking sections is not viable, would be required to become smoke-free. Thus, many non-smokers and employees in smaller public venues will still be exposed to harm from SHS.

Secondly, the owners of larger public venues (more than 70 square metres) are required to formulate designated smoking areas of up to 30 per cent of the total usable space. A last-minute modification to the new law just days before implementation even allows smoking in public venues larger than 300 square metres, provided that the smoking area does not exceed 40 per cent of the total space of the venue. It is unclear why a partial smoking ban is preferred over a total ban in larger establishments, when the available evidence suggests that partial bans do not reduce SHS exposure effectively. Furthermore, as our own research has shown, observing smoking in public places increases the risk for smoking uptake in Greek adolescents, and conveys the message that smoking is the norm.

The law requires smoking and non-smoking areas to be separated with a floor to ceiling glass partition, and to prohibit entry to smoking areas by adolescents younger than 18 years. However, separating smoking from non-smoking areas with glass, as the new law demands, does not prevent or reduce observation of smoking in public venues, even if adolescents are not allowed to enter the non-smoking area. This, together with smoking still being allowed in smaller premises, may reduce or negate the potential of the new law to change societal norms that encourage smoking initiation among young people.

Finally, we have found that more than half of a sample of Greek smokers reported that they had smoked in non-smoking public areas. A partial smoking ban can hardly help to reduce such non-compliance, because it can create ambiguities and lead to misconceptions regarding the areas where smoking is actually allowed. Hence, the new law may actually encourage non-compliance, in addition to preserving the norm of smoking being part of social life.

The tobacco industry has long been well aware of the impact of comprehensive smoking bans in public places. As one Philip Morris document put it in 1993, “When smoking restrictions in the workplace, public transport and restaurants are implemented, enforced and respected, demand for cigarettes decreases and incidence among adult smokers falls...” Our concern is that the exceptions and ambiguities in the new Greek legislation may severely weaken the consistency with which the restrictions are implemented, enforced and respected. (See also Vardavas CI & Behrakis P. Greece: action at last! *Tob Control* 2009;**18**;78–81)

LAMBROS LAZURAS, AGGELOS RODAFINOS

Thessaloniki, Greece.

llazuras@seerc.org

RICHARD EISER

University of Sheffield, UK

## KENYA: BEATING CASE FOR COURT

The Kenya Tobacco Control Alliance (KTCA) has alleged that one of its members, George Kivanda, was beaten up by a local manager of Alliance One Tobacco, a company working under contract to British American Tobacco (BAT) in a tobacco-growing area. KTCA says that the manager appears to have taken exception to Mr Kivanda trying to speak to journalists who wanted to report on tobacco farmers’ conditions. The alleged offence took place in the Migori district of Nyanza province, in the south west of the country. Lawyers acting for Mr Kivanda duly brought a court case and after a preliminary hearing, in which the Alliance One manager denied the charges against him, a full hearing is expected later in the year.

**Figure:**
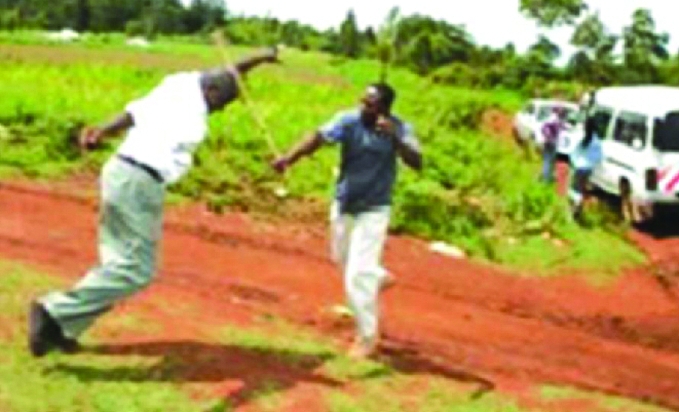
Kenya: a photo showing the alleged assault on a tobacco control advocate.

## CANADA: QUIET HEROINE REMEMBERED

Henry Fonda had the ability to imbue the common man with greatness. As Tom Joad in The Grapes of Wrath or as a juror in Twelve Angry Men, Fonda’s soft-spoken dignity compelled others to listen, inspiring them ultimately to perform their own acts of bravery. But Fonda employed an actor’s craft to achieve his ends. An equally quiet-spoken leader, Heather Crowe, achieved greatness and fashioned a legacy more enduring and valuable than anything conceived in Hollywood. Last month, the city of Ottawa, the capital of Canada where Crowe lived, worked and died, inaugurated a lasting memorial to her achievements.

Heather Crowe was a hardworking, low wage-earning waitress, putting in long hours over a period of 40 years in Ottawa restaurants where “the air was blue with cigarette smoke.” A lifelong non-smoker, Crowe was diagnosed with lung cancer due to her prolonged second-hand smoke exposure. Not quite 57 at the time, in 2002, she would only live only four more years after her Stage 3 diagnosis, dying at 61, losing what could have been many rich years, even decades, with her daughter, grand-daughter, family and friends.

For so many people faced with cancer, particularly one with a poor prognosis, there is a tendency to retreat from the wider world, taking refuge in the privacy, comfort and love of family. And yet, Heather Crowe, the quintessential face in the crowd, a well-liked waitress but unknown outside the circles of work and family, would change the face of public health in Canada.

Determined to be “the last person to die from second-hand smoke at work,” Crowe became an effective advocate, lobbying politicians first in Ottawa, the nation's capital, then across the province of Ontario and the entire country. She challenged the Ontario Workplace Safety and Insurance Board and won her precedent-setting claim to have her second-hand smoke-induced illness as a work-related form of injury. Although she would die just before it passed, she was instrumental in the development of the Smoke Free Ontario Act. Working with Physicians for a Smoke-Free Canada, she energised community groups and, in particular, young people across the country and left a series of new or improved municipal bylaws in her wake.

Heather Crowe literally became the face of second-hand smoke, collaborating with Health Canada on a provocative, compelling set of TV and print ads. And the message she repeated was this: “Anyone who doesn’t think second-hand smoke kills can just ask me... I am the canary in the coal mine for the hospitality industry.”

On May 22nd, 2009, three years to the day since her death, the city of Ottawa dedicated a public park to Heather Crowe’s memory. The politicians spoke, as she once did, in simple, eloquent terms. But the greatest tribute was to designate the site the first non-smoking park in Ottawa. In death as in life, Heather Crowe set precedents, touched lives and left a vibrant legacy.

**Figure:**
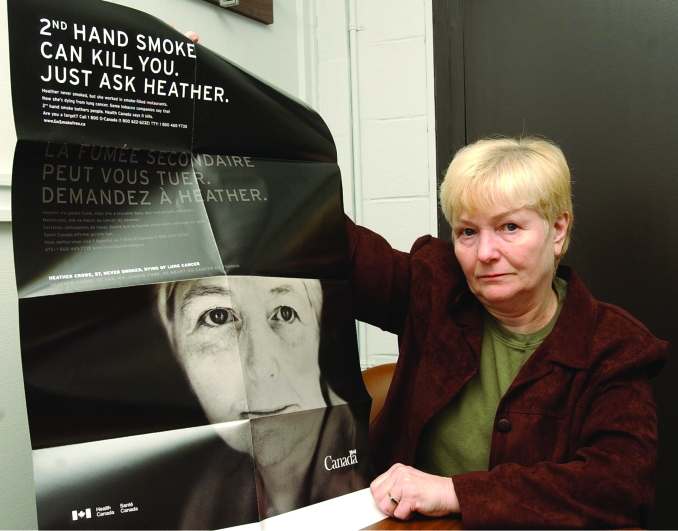
Canada: Heather Crowe pictured with one of the health education posters bearing her photograph and message, which she helped the Canadian health ministry to devise.

STAN SHATENSTEIN

Associate news editor Montreal, Canada

shatensteins@sympatico.ca

